# Establishment of a Suitable Diagnostic Workflow to Ensure Sensitive Detection of African Swine Fever Virus Genome in Porcine Semen

**DOI:** 10.3390/pathogens13070537

**Published:** 2024-06-25

**Authors:** Virginia Friedrichs, Darwin Reicks, Jeffrey J. Zimmerman, Eric A. Nelson, Carola Sauter-Louis, Martin Beer, Jane Christopher-Hennings, Sandra Blome

**Affiliations:** 1 Friedrich-Loeffler-Institut, Suedufer 10, 17493 Greifswald-Insel Riems, Germany; virginia.friedrichs@fli.de (V.F.); carola.sauter-louis@fli.de (C.S.-L.); martin.beer@fli.de (M.B.); 2 Reicks Veterinary Research and Consulting, Saint Peter, MN 56082, USA; darwin@rvrcmn.com; 3 Veterinary Diagnostic & Production Animal Medicine, Iowa State University, Ames, IA 50011, USA; jjzimm@iastate.edu; 4 Animal Disease Research & Diagnostic Laboratory, South Dakota State University, Brookings, SD 57007, USA; eric.nelson@sdstate.edu

**Keywords:** African swine fever virus, virus diagnostics, boar semen, commercial qPCR kits, comparison, nucleic acid extraction, sensitivity, performance

## Abstract

The rapid spread of African swine fever virus (ASFV), causing severe and often lethal disease in domestic pigs and Eurasian wild boar, continues to be a threat to pig populations and dependent industries. Despite scientific achievements that have deepened our understanding of ASFV pathogenesis, alternative transmission routes for ASFV remain to be elucidated. We previously demonstrated the efficient transmission of ASFV from infected boars to naïve recipient gilts via artificial insemination, thereby highlighting the importance of surveillance of boar semen prior to its shipment. Since the accurate and reliable detection of even low amounts of ASFV in boar semen is key to disease prevention and control, we established a suitable diagnostic workflow to efficiently detect the ASFV genome in boar semen. Here, we assessed the sensitivity of various routine nucleic acid extraction kits as well as qPCR protocols in detecting the ASFV genome in the blood and semen of infected boars. The feasibility of the respective kits and methods for future use in boar studs was also considered. Variability in sensitivity mostly concerned samples with low to very low amounts of the ASFV genome. Ultimately, we defined a well-suited workflow for precisely detecting the ASFV genome in boar semen as early as 2 days post ASFV infection.

## 1. Introduction

The rapid spread of African swine fever virus (ASFV), an enveloped, double-stranded DNA virus with a genome of 170–190 kpb, remains a threat to pig populations and economies worldwide [1,2]. Originally, ASFV circulated in an ancient sylvatic cycle among asymptomatic warthog and soft tick (genus: *Ornithodoros*) populations in sub-Saharan Africa [3]. Its introduction into domestic pigs or Eurasian wild boar, however, leads to severe yet rather unspecific clinical signs, resulting in high case fatality rates [4,5]. The disease is notifiable to the World Organization for Animal Health (WOAH). The current African swine fever (ASF) panzootic started in 2007, when ASFV was introduced into Georgia and subsequently into the Russian Federation and many Trans-Caucasian countries. In 2014, ASF entered the European Union [6], in 2018, China [7], and in 2021, the Caribbean [8].

At present, 24 different genotypes, characterized based on variations within the p72 capsid protein encoded by the B464L gene, have been defined [9,10,11,12]. However, only genotype I and II strains have been found outside of Africa, and the current panzootic involves genotype II strains only. Despite efforts to understand and restrict the disease, its ongoing spread emphasizes the need to evaluate alternative transmission routes and strengthen early warning systems. In a previous study, we demonstrated that artificial insemination is an efficient route to transmit ASFV from infected boars to naïve recipient gilts. Usually, domestic boar semen originates, with the exception of rural backyard farming, from boar studs [13]. In these facilities, boars are kept individually, and semen is collected regularly on demand. The collected semen has to pass mandatory quality control checks, e.g., count of spermatozoa, amount of abnormalities, and mobility. Since we showed that none of those criteria were affected by early ASFV infection or even acute viraemia, risk-based surveillance for the presence of ASFV in boar semen is of the utmost importance and opens up the only possibility for very early detection. Around the globe, real-time polymerase chain reaction (qPCR) is widely used as a reliable, sensitive, and specific tool for detecting animal diseases such as ASFV [14,15,16,17]. In addition to a robust qPCR system, a highly effective extraction method is key to the correct laboratory diagnosis [18]. Recommended extraction systems are listed in the WOAH guidelines [19].

However, the extraction methods and qPCR assays need to be constantly adapted to and meet putative evolutionary changes in the virus and/or the demands of the diagnostic field. This is especially true for such a difficult matrix as semen. In recent years, several commercially available qPCR kits for ASFV genome detection have become available, and performance comparisons have been carried out, e.g., Schoder et al. (2020) [20] and Pikalo et al. (2022) [19]. To standardize the conditions and facilitate data interpretation, regional reference laboratories should follow the guidelines provided by the WOAH and regional (e.g., the European Union) and national reference laboratories (NRLs). These guidelines include a list of registered or licensed diagnostic methods suited to and permitted for routine diagnostics [21].

As semen preparations for artificial insemination contain potentially qPCR-inhibitory components [22], such as sucrose [23], they represent complex and highly challenging matrices for routine diagnostic testing, also affecting ASFV diagnostic workflows. Hence, it is of the utmost importance to define a suitable diagnostic workflow to accurately detect the ASFV genome in boar semen.

Here, we compared three methods for nucleic acid extraction, as well as five qPCR protocols, to establish a suitable workflow for the efficient and early detection of the ASFV genome in boar semen using a standard methodology without special semen treatment. The criteria applied for definition were (I) reliable amplification, (II) handling and time requirements, and (III) efficient detection of the ASFV genome in critical samples, e.g., samples obtained early after inoculation, especially those presumed to contain only very few ASFV genome copies.

## 2. Materials and Methods

### 2.1. Samples

All samples used in this study were derived from breeding boars, which were intramuscularly inoculated with 10^4^ HAD_50_ of the ASFV strain “Estonia 2014” [24]. This trial included four adult breeding boars, two Large White and two Pietrain boars [25]. Semen samples from all boars were collected regularly after experimental ASFV infection to enable early ASFV screening in porcine semen. Since EDTA blood, already tested for accurate and early detection of the ASFV genome [26], was previously defined as the “gold standard” matrix, blood samples were compared in terms of analytical performance with the semen samples. Positive EDTA blood (*n* = 18) and semen samples (*n* = 17) obtained at days 2, 3, 4, 5, 14, and 20 post inoculation were included. Table 1 provides details on the semen samples included in the study. The EDTA blood samples corresponded to these semen samples.

### 2.2. Extraction of Viral DNA

All the samples were frozen at −80 °C upon collection to ensure full availability of the cell-bound viral genome [27]. For accurate detection of low ASFV genome loads in the boar semen, the performance of three commercially available, routinely used nucleic acid extraction kits was compared (Table 2): (I) the NucleoMag^®^ VET kit (Macherey-Nagel, Düren, Germany); (II) the MagMAX™ Pathogen RNA/DNA kit (Thermo Fisher, Waltham, MA, USA); and (III) the MagMAX™ 96 Viral RNA isolation kit (Thermo Fisher, Waltham, MA, USA). All the samples were extracted in triplicate (*n* = 3), and all kits were used according to the manufacturer’s instructions unless stated otherwise. Extraction was performed on the automated extraction platform KingFisher™ 96 flex (Thermo Fisher, Darmstadt, Germany) upon utilization of the extraction protocols provided by the manufacturers.

Three criteria were used to define the sensitivity of the assay and ultimately the most appropriate extraction method: (I) sensitivity across all “true positive” samples, (II) sensitivity based on the sample set taken early after inoculation, i.e., 2–3 dpi, and (III) preference for lower Cq values.

Because early detection of the ASFV genome in semen is crucial, the semen samples were divided into “early” (2–3 dpi, *n* = 5 positive samples) and “late” samples (>4 dpi, *n* = 9 positive samples) prior to performance assessment. True positive samples among the “early” samples were defined according to manual extraction and increasing the number of replicates, which was beneficial for the detection of samples with low amounts of the ASFV genome. For manual extraction, DNA from 85 µL of semen was extracted using the QIAamp Viral RNA Mini kit (QIAGEN). Manual extraction was performed in triplicate (*n* = 3), and the samples were analyzed in three independent qPCR runs (using the VetAlert™ ASFV DNA Test Kit from Tetracore, Rockville, MD, USA), where each sample was measured in triplicate (*n* = 3).

### 2.3. Molecular Assays: qPCR

Following extraction, all the samples were compared using the VetAlert ASFV DNA assay (Tetracore). This assay is accredited in the German national reference laboratory (NRL) and was among the best commercial qPCR kits in previous studies when using various other sample matrices from ASFV-infected pigs [19]. The best-performing extraction method was subsequently used to assess the analytical performance of the five qPCR assays in ASFV detection (all certified for use in ASFV diagnostics).

The five qPCR protocols included in this study were (Table 3) the (I) VetAlert™ ASFV DNA Test Kit; (II) the virotype ASFV 2.0 PCR Kit (Indical); (III) the VetMax™ ASFV Detection Kit (Thermo Fisher), (IV) the WOAH-recommended protocol published by King et al. (2003) [15] with slight modifications (accredited ASF System 1); and (V) the RealPCR ASFV DNA Test (IDEXX). All protocols were utilized according to the manufacturer’s/authors’ instructions.

To facilitate the detection of low ASFV genome copy numbers in the semen samples, the above-mentioned extracted DNA from the semen samples (*n* = 3 per boar and time point) was evaluated in triplicate (a total of *n* = 9 per semen sample) for each qPCR assay. For the qPCR assays, the same sensitivity criteria as used for the extraction methods were employed.

### 2.4. Data Analyses

All the data generated by qPCR were visualized and analyzed for statistical relevance using GraphPad Prism 9 (GraphPad Software Inc.). Statistical analyses were performed using One-Way ANOVA with Tukey’s post hoc testing, and significant differences are depicted as follows: ** *p* < 0.01, *** *p* < 0.001, **** *p* < 0.0001. Subsequently, all the qPCR test results were compared to the best-performing protocol using the Bland–Altman test [28]. The optimal performance was defined as the number of wells with successful detection of the ASFV genome in semen samples 2–3 dpi. The Limit of Agreement (LoA) interval for each comparison was defined as the mean difference ±1.96 standard deviation (SD) of the Cq values. Furthermore, the percentage of positive results among the infected boars (true positive and false negative) was calculated for each qPCR method/matrix. Additionally, using samples from 2 and 3 dpi, a repeated-measures ANOVA, correcting for replicates, was performed to test for statistical differences in the performance of the five qPCR tests. However, the power of the statistical calculations was limited because correlated samples were used (repeated sampling of a few individuals). Due to many samples not yielding Cq values upon analysis, the number of cycles minus Cq values was used for calculations. However, the power of the statistical calculations was limited since data for only a few animals were available.

## 3. Results

### 3.1. Assessment of Preparation Time, Handling, and Time Requirements

#### 3.1.1. Nucleic Acid Extraction Kits

The sample preparation time, which correlates directly with the number of mandatory pipetting steps, varied significantly between manufacturers. For example, the NucleoMag^®^ VET kit only required the sample to be vortexed prior to adding the ready-to-use lysis buffer, while the MagMAX™ Pathogen RNA/DNA Kit called for lysis buffer and bead mix preparation (2 and 3 steps, respectively) before DNA extraction. Differences were also found between matrices: EDTA blood could be added to the prepared solutions, while the semen samples required preparation of the lysate in a separate plate. The subsequent semen lysate was then added to the plate containing all the necessary washing/elution solutions. An additional difference was notable for the MagMAX™-96 Viral RNA Isolation Kit, which included the preparation of the lysis/binding buffer and bead mix; however, the EDTA blood and semen samples did not require varying preparation steps.

However, the steps required for nucleic acid extraction were comparable between all kits, as listed in Table 1.

In terms of storage, all the kits contained components that needed to be stored at different temperatures (−20 °C/4 °C/RT for kits by Thermo Fisher, −20 °C/RT for the NucleoMag^®^ VET kit), and all kits allowed for the use of automated nucleic acid extraction platforms.

#### 3.1.2. Differences Amongst Tested ASFV qPCR Assays

The required pipetting steps during the preparation of the qPCR reactions overall ranged from two to five steps (Table 2). Two out of five kits included a ready-to-use mix that only needed mixing with the respective sample in the plate: the virotype ASFV 2.0 PCR Kit and the VetMax™ ASFV Detection Kit. Furthermore, the VetAlert™ ASFV DNA Test Kit needed one extra step: adding the enzyme to the mix. Two methods, the WOAH King et al. protocol and the RealPCR ASFV DNA Test kit, required step-by-step mixing of all reagents, with five and three steps, respectively [15].

In terms of the qPCR cycles for target amplification, all assays included similar cycles, i.e., 45, except for the virotype ASFV 2.0 PCR assay, which runs with 40 cycles. The duration of the qPCR runs was also heterogenous, ranging from 1 h and 2 min (virotype ASFV 2.0 PCR Kit) to 2 h and 25 min (WOAH King et al. [15]).

### 3.2. Extraction Efficiency Evaluation of the ASFV Genome from Boar Semen

**Figure 1:**
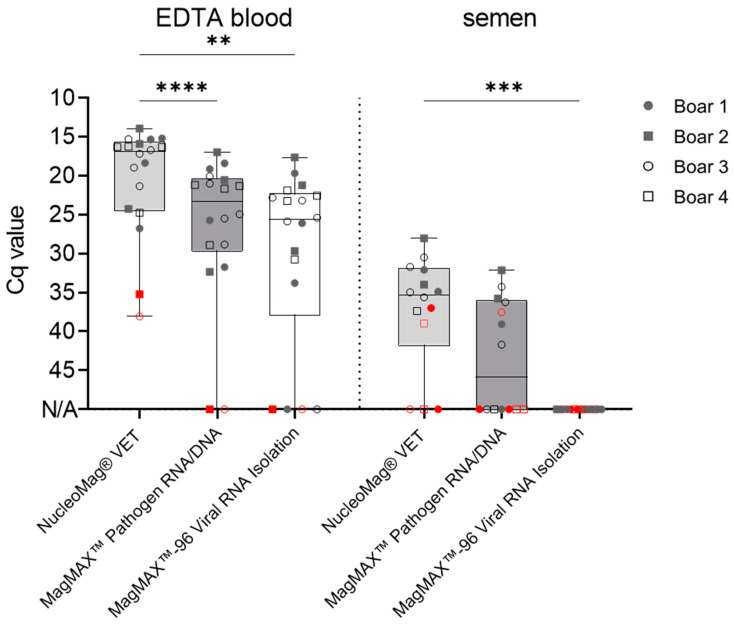
Comparison of ASFV genome extraction efficiency from boar blood and semen. Evaluation of performance using 18 ASFV-positive blood and 14 positive semen samples. Genome loads were evaluated by qPCR with the VetAlert™ ASFV DNA Test Kit. Boxes represent 25/75 percentiles, including the group median with min. and max. values; each individual is represented by a symbol. Critical EDTA blood (2 dpi) and semen (2–3 dpi) samples are indicated in red. All samples were evaluated in triplicate (*n* = 3). N/A = no detection occurred within 45 cycles. Significant differences were assessed by One-Way ANOVA, ** *p* < 0.01, *** *p* < 0.001, **** *p* < 0.0001.

As shown in Figure 1, sample extraction using the NucleoMag^®^ VET Kit resulted in 100% detection of positive EDTA blood samples (*n* = 18/18) and 78.6% detection (*n* = 11/14) of the infected semen samples. Furthermore, the MagMAX™ Pathogen RNA/DNA Kit achieved detection of up to 88.9% (*n* = 16/18) and 50.0% (*n* = 7/14) of the positive EDTA blood and semen samples, respectively. Similar results were obtained for the EDTA blood samples (88.9%; *n* = 16/18) extracted with the MagMAX™-96 Viral RNA Isolation Kit; however, no semen samples extracted with this kit yielded Cq values when analyzed with the VetAlert™ ASFV DNA assay (Figure 1). The extraction of semen samples using the MagMAX™-96 Viral RNA Isolation Kit was carried out three times, further verifying these results.

Out of fourteen positive semen samples, five were positive at an early time point (2–3 dpi). Extraction with the NucleoMag^®^ VET Kit detected 40.0% (*n* = 2/5) of the true positive samples, followed by extraction with the MagMAX™ Pathogen RNA/DNA Kit (*n* = 1/5).

Differences in the subsequent Cq values under the same cycling conditions were also observed between the extraction kits. Extraction with the NucleoMag^®^ VET Kit rendered the lowest Cq values (EDTA blood: 20.3 ± 7 SD; Semen: 34.1 ± 3.3 SD), followed by the MagMAX™ Pathogen RNA/DNA Kit (Blood: 23.6 ± 4.8 SD; Semen: 36.7 ± 3.1 SD) and the MagMAX™-96 Viral RNA Isolation Kit (Blood: 24.6 ± 4.4 SD), as shown in Figure 1. Samples with low amounts of the ASFV genome, e.g., the blood samples of boars #2 (Cq 35.2 ± 0.2 SD) and #3 (Cq 38.1 ± 4.5 SD) at 2 dpi, were not accurately detected after extraction with the MagMAX™ kits.

### 3.3. Assessment of qPCR Performance in Detecting the ASFV Genome in Boar Semen

Based on these results, the most suitable routine kit for the extraction of viral genome copies from the semen samples with an automated platform was the NucleoMag^®^ VET Kit. Therefore, a comparison of the analytical performance of the five qPCR assays was carried out using nucleic acids extracted via this method.

**Figure 2:**
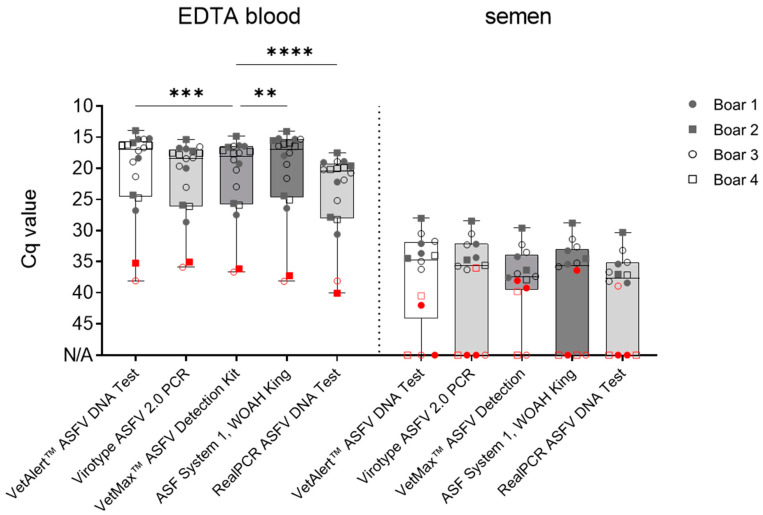
Analytical sensitivity for boar blood and semen in qPCR. Evaluation of qPCR protocol performance, utilizing 18 ASFV-positive blood and 14 positive semen samples. Boxes represent 25/75 percentiles, including the group median with min. and max. values; each individual is represented by a symbol. Critical EDTA blood (boar #2 and #3 at 2 dpi) and semen (2–3 dpi) samples are indicated in red. Samples were evaluated in triplicate (*n* = 3). N/A = no detection occurred within 45 cycles. Significant differences were assessed by One-Way ANOVA, ** *p* < 0.01, *** *p* < 0.001, **** *p* < 0.0001.

Differences amongst matrices were observed in all the included assays. All positive EDTA blood samples (*n* = 18) were detected by all assays (100% sensitivity); however, differences in the Cq values were noted. The VetAlert™ ASFV DNA assay yielded similar values (20.3 ± 7 SD) to ASF System 1, recommended by WOAH, King et al. [15] (20.4 ± 7.3 SD). Following this, the virotype ASFV 2.0 (21.5 ± 6.3 SD) and VetMax™ ASFV Detection assays (21.3 ± 6.6 SD) rendered similar Cq values. Finally, the RealPCR ASFV DNA Test (23.8 ± 6.6 SD) showed higher Cq values for analogous samples (Figure 2).

For the second matrix, semen, all the mean Cq values were comparable and ranged from 33.3 ± 2.6 SD (virotype ASFV 2.0 PCR Kit) to 35.7 ± 2.6 SD (RealPCR ASFV DNA Test), as depicted in Figure 2**.**

However, when the samples were divided into “early” and “late” collected samples, the results were more heterogenous (Figure 2, Table 4). All the qPCR assays successfully detected the ASFV genome in “late” boar semen samples obtained at 4−20 dpi (*n* = 9). Nonetheless, differences were noted for the “early” semen samples (2−3 dpi; *n* = 5 true positive). Based on this sample set alone, the VetMax™ ASFV Detection Kit was able to detect 60% of all the true positive early samples (*n* = 3 with Cq 38.1 ± 2.6 SD), followed by the VetAlert™ ASFV DNA Test Kit, which detected 40% (*n* = 2 with Cq 39.6 ± 1.8 SD). Finally, ASF System 1 (Cq 33.6 ± 0.8 SD), the virotype ASFV 2.0 PCR Kit (Cq 36), and the RealPCR ASFV DNA Test (Cq 38.9 ± 1.2 SD) were able to detect 20% (*n* = 1) of the early semen samples. If sensitivity is calculated across all semen samples, the VetMax™ ASFV Detection Kit shows a sensitivity of 85.7% (*n* = 12/14), the VetAlert™ ASFV DNA Kit a sensitivity of 78.6% (*n* = 11/14), and the other kits a sensitivity of 71.4% (ASF-System 1, virotype ASFV 2.0 PCR, and RealPCR ASFV DNA Test, all *n* = 10/14).

**Figure 3:**
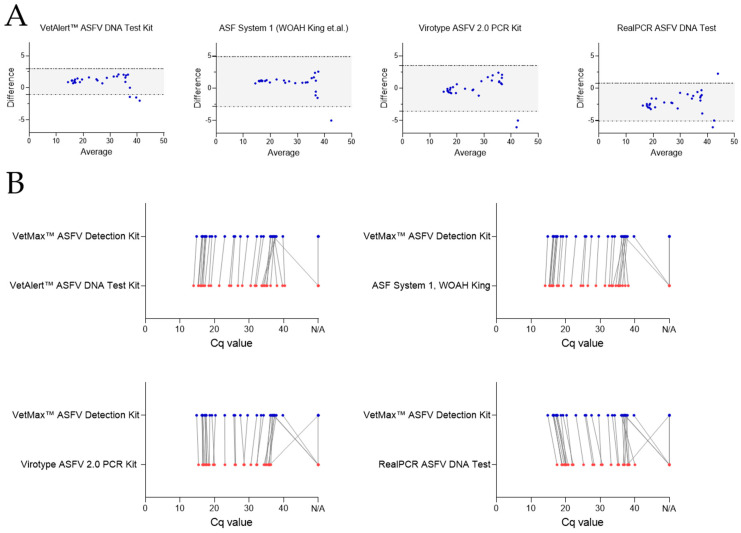
Comparison of five qPCR assays in ASFV genome detection efficiency from boar blood and semen. Evaluation of qPCR method performance, utilizing 18 ASFV-positive blood and 14 positive semen samples. All qPCR kits were compared to the VetMax™ ASFV Detection Kit. (**A**) Bland–Altman plots. Gray areas represent the lower and upper limits of agreement. Blue points display differences between the VetMax™ ASFV Detection Kit and any other qPCR method tested (WOAH King et al. [15]). (**B**) Point-by-point result comparison of all samples tested in relation to the VetMax™ ASFV Detection Kit. N/A = no detection occurred within 40–45 cycles. Lines connect results of corresponding samples.

The analytical performance of the qPCR kits using all the true positive EDTA blood and semen samples (*n* = 32) was assessed using Bland–Altman plots (Figure 3A) and point-by-point evaluations (Figure 3B). The best-performing assay, the VetMax™ ASFV Detection Kit, was used as a reference for the point-by-point evaluations.

The VetAlert™ ASFV DNA Test Kit (bias 0.97) showed a narrow LoA, indicating a high level of agreement. However, three samples were found outside the LoA, which means that the results of these three samples varied beyond the calculated standard deviation. Two assays, ASF System 1 and the virotype ASFV 2.0 PCR Kit, showed a similar LoA, whereas one and two samples, respectively, were underestimated with these systems when compared with the VetMax™ ASFV Detection Kit. Finally, the RealPCR ASFV DNA Test kit showed samples with Cq values that were both under- and overestimated, indicating higher variability and therefore disagreement in the results between these kits. A manual in-detail comparison revealed once more that these semen samples were detected with a distinct shift in Cq values for the extraction of the ASFV genome. This indicates that out of the tested assays, only the VetAlert™ ASFV DNA Test Kit can be used interchangeably with the best-performing kit, the VetMax™ ASFV Detection Kit. The point-by-point comparison revealed that the VetAlert™ ASFV DNA Test Kit overall had lower Cq values up to a Cq of ~37, where the detection of weakly positive samples was superior using the VetMax™ ASFV Detection Kit (Figure 3B). The virotype ASFV 2.0 PCR Kit and ASF System 1 had comparable Cq values to the VetMax™ ASFV Detection Kit for the EDTA blood samples but failed to detect most of the weakly positive semen samples. The RealPCR ASFV DNA Test had overall higher Cq values but also failed to detect the weakly positive semen samples.

**Figure 4:**
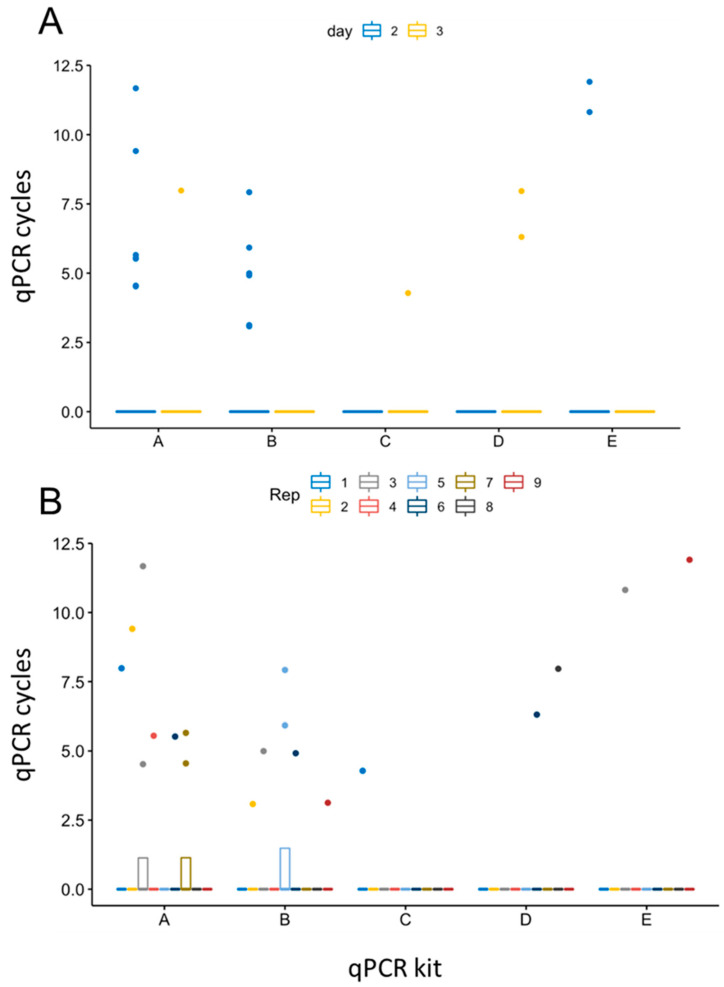
Evaluation of the detection accuracy of five qPCR kits using semen samples of infected boars. Repeated-measures ANOVA was performed to calculate significant differences in detection performance of the five qPCR kits. Variance between 2 and 3 dpi (**A**), as well as among replicates of each sample (**B**) (*n* = 8 with 9 replicates each), was calculated. X-axis labeling corresponds to qPCR kits: A = VetMax™ ASFV Detection Kit, B = VetAlert™ ASFV DNA assay, C = virotype ASFV 2.0 PCR assay, D = RealPCR ASFV DNA Test, E = ASF System 1 (WOAH King et al. [15]).

Furthermore, repeated-measures ANOVA resulted in a statistically significant difference in the detection efficiency of the five different PCRs using semen samples from 2 and 3 dpi (*p* = 0.0068, Figure 4). While the calculations revealed statistical significance between 2 and 3 dpi (*p* = 0.0042, Figure 4A), no significant variations were observed among the replicates of each sample (*n* = 8 samples, each 9 replicates, Figure 4B). Of the two-way interactions, significance was confirmed between the results of the five qPCR kits (*p* = 0.0087), indicating their differing efficiency in detecting samples with low amounts of the ASFV genome correctly.

## 4. Discussion

As broadly protective vaccinations or reliable treatment options for ASF are still not available, the accurate and early identification of infected individuals is key to prevent further spread of the disease. The modern pork industry mainly relies on artificial insemination, with the boar semen acquired from boar studs. The semen is collected, diluted with nutrient-containing extenders, and shipped on demand, often nationwide or even across borders. To prevent the spread of ASFV-containing semen, surveillance of semen upon collection is needed. However, fast processing during quality management is essential to ensure the high quality and viability of the spermatozoa. Therefore, we compared various nucleic acid extraction kits, as well as established and validated ASFV-specific qPCR assays, to define a practicable diagnostic workflow without the need for special treatments or protocols for the early detection of even low amounts of the ASFV genome in boar semen in a high-throughput diagnostic laboratory. Similar studies were carried out comparing the performance of WOAH-approved qPCR assays on wild boar samples of varying degrees of decay [19]. Here, the best-suited kits were defined according to the following criteria: (I) sensitivity across the full sample set, (II) sensitivity based on semen samples obtained on days 2 and 3 after inoculation, and (III) yield of low Cq values. Since fresh boar semen typically is shipped less than 24 h after collection, the sample preparation and qPCR run duration served as additional factors for efficiency determination.

Generally, no combination of nucleic acid extraction and qPCR kit was able to detect all the true positive semen samples, as defined by manual DNA extraction. However, regarding the nucleic acid extraction kits, considerable differences in their efficiency in extracting the ASFV genome from boar blood and semen were noted. While extraction with the NucleoMag^®^ VET Kit resulted in the detection of all the true positive blood samples, MagMAX™ kits gave two false negative results for the two samples with the lowest ASFV genome loads (boar #2 and #3 at 2 dpi). Furthermore, the differences were even much more striking regarding semen. Here, extraction with the NucleoMag^®^ VET Kit resulted in 11/14 true positive results, while extraction with the MagMAX™ Pathogen RNA/DNA Kit obtained 7/14 true positive results. However, repeated extraction with the MagMAX™-96 Viral RNA Isolation Kit did not yield any positive results for semen. Conclusively, while their performance on blood samples was largely comparable, the extraction efficiency varied between the kits. Overall, reduced extraction efficiency for semen was noted for all the extraction kits included in this study, indicating the presence of inhibitory components in this matrix, e.g., polysaccharides [23,29]. Additionally, low amounts of the genome in the semen early after infection might result in false negative results, considering that only a tiny fraction of the whole ejaculate is sampled. Hence, detection variability is likely to occur in samples with low amounts of the target genome. Based on the detection efficiency in our study and the short preparation time, the NucleoMag^®^ VET Kit seems well suited to facilitate ASFV monitoring in boar studs. Although the manual extraction of DNA is considered the most sensitive method for ASFV diagnostics, an automated platform with a similar detection efficiency is likely more suitable for monitoring large pig herds. Given that we focused on routine diagnostic workflows with a high throughput, we did not include special extraction protocols for semen (as can be found in the WOAH manual for, e.g., Bovine herpesvirus 1 infection: https://www.woah.org/fileadmin/Home/eng/Health_stanards/tahm/3.04.11_IBR_IPV.pdf (accessed on 6 May 2024)).

Additionally, similar observations were made for the studied qPCR assays. By extracting nucleic acids via the NucleoMag^®^ VET Kit, all the qPCR methods successfully detected all the true positive EDTA blood samples, even samples containing low amounts of the ASFV genome, e.g., boars #2 and #3 at 2 dpi. This further indicated the unmatched suitability of blood samples for the accurate and early detection of the ASFV genome in pigs, as described previously [26]. It is of note that the boars tolerated blood sampling through their saphenous veins without becoming agitated during semen collection. Hence, the collection of small amounts of blood during the procedure might be feasible for obtaining blood for ASFV surveillance, as already routinely applied for PRRSV [30,31]. However, although the Cq values of the EDTA blood samples were largely comparable, they were significantly higher using the RealPCR ASFV DNA Test, suggesting that there could be an impact on the detection of low-positivity samples. Furthermore, although the VetAlert™ ASFV DNA Test Kit and ASF System 1 rendered lower Cq values in general, the Cq values of the weakly positive samples attained with the VetMax™ ASFV Detection Kit were lower, indicating increased performance with weakly/very weakly positive samples as the input matrix. Detection in semen benefited from increasing the number of replicates in qPCR, as described previously [32].

Although no qPCR assay was able to detect all the true positive samples (EDTA blood and semen taken together), the VetMax™ ASFV Detection Kit rendered (I) the most positive results (3/5) and (II) overall lower Cq values, which reached statistical significance for the EDTA blood samples, further enhancing the suitability of this kit for accurate ASFV detection. It is of note that in-depth analyses of the kit performances uncovered that the VetAlert™ ASFV DNA Test Kit can be used interchangeably with the VetMax™ ASFV Detection Kit due to their similar performance. However, in terms of the handling/preparation time and complexity, the VetMax™ ASFV Detection Kit provided a ready-to-use mix, while the VetAlert™ ASFV DNA Test Kit required the preparation of said mix.

In addition, the virotype ASFV 2.0 PCR Kit only detected 1/5 true positive semen samples, which possibly resulted from the number of amplification cycles recommended for this kit. Considering that the Cq values of true positive semen samples can exceed 40, the virotype ASFV 2.0 PCR Kit was likely at a disadvantage due to being optimized for time (with the shortest run among all tested), not sensitivity.

In summary, based on our dataset, we identified the NucleoMag^®^ VET Kit as the most suitable kit for nucleic acid extraction to enable the detection of even low amounts of the ASFV genome in porcine blood and semen samples. Furthermore, the VetMax™ ASFV Detection Kit and, although to a lesser extent, the VetAlert™ ASFV DNA Test Kit provided paramount detection of weakly positive blood and semen samples among all the kits tested. However, the suitability for diagnostic workflows of each kit must be carefully assessed, e.g., handling, the possibility of combining assays, the time needed, and the range of detectable pathogens with kits from one manufacturer. This is especially true when samples with expectedly high Cq values (obtained during the early stages of infection without apparent clinical signs) are handled.

## 5. Conclusions

With this study, we present a suitable workflow that enables the efficient detection of the ASFV genome in boar semen using routine protocols. We compared the performance of three widely used magnetic-bead-based methods for nucleic acid extraction, as well as WOAH-recommended qPCR methods and commercial qPCR kits. Among the tested options and based on our dataset, the NucleoMag^®^ VET Kit performed best, in combination with the VetMax™ ASFV Detection Kit. Nevertheless, the limitations of this study must be considered, as the sample sizes were small (blood *n* = 18, semen *n* = 17) and the samples were correlated, as they were derived from four individuals rather than being independent.

## Figures and Tables

**Table 1:** Semen samples included in the study. The ASF status as true positive (POS) or negative (NEG) was based on manual extraction using the QIAamp Viral RNA Mini kit (QIAGEN) in triplicate, followed by the VetAlert™ PCR as a reference (three independent runs on all replicates). dpi = days post inoculation; NT = not tested.

dpi	Boar #1	Boar #2	Boar #3	Boar #4	POS/Total
2	**POS**	NEG	NEG	**POS**	**2**/4
3	**POS**	NEG	**POS**	**POS**	**3**/4
4	**POS**	**POS**	**POS**	**POS**	**4**/4
5	**POS**	**POS**	**POS**	NT	**3**/3
14	NT	NT	**POS**	NT	**1**/1
20	NT	NT	**POS**	NT	**1**/1
					***n* = 14/**17

**Table 2:** Specifications of all nucleic acid extraction kits used to extract DNA from blood and semen.

Extraction Kit Name	Input Volume [µL]	Output Volume [µL]	Steps
NucleoMag^®^ VET	100	100	8
MagMAX™ Pathogen RNA/DNA Kit	100 bl/115 se	90	7 bl/8 se
MagMAX™-96 Viral RNA Isolation Kit	50	90	9

Legend: bl = blood samples; se = semen samples.

**Table 3:** Specifications of all ASFV detection (qPCR) protocols and kits used in this study.

qPCR Kit Name	Input Volume [µL]	Internal Control	Cycles	Pipetting Steps/Time
VetAlert™ ASFV DNA Test Kit	5	exogenous	45	3/1 h 36 min
virotype ASFV 2.0 PCR Kit	5	exo-/endogenous	40	2/1 h 2 min
VetMax™ ASFV Detection Kit	5	exogenous	45	2/1 h 18 min
ASF System 1 (WOAH, King et al. [15])	5	endogenous	45	5/2 h 25 min
RealPCR ASFV DNA Test	5	endogenous	45	4/1 h 30 min

**Table 4:** Overview of successfully detected true positive samples among critical semen samples (2–3 dpi). To account for statistical effects, samples were tested in nine replicates in total (three extraction replicates in three PCR runs).

Kit Name	dpi	No. of Positive Replicates			
		Boar 1	Boar 2	Boar 3	Boar 4
**VetAlert™ ASFV DNA**	2	**3/9**	—	—	**2/9**
	3	0/9	—	0/9	0/9
virotype ASFV 2.0 PCR	2	0/9	—	—	0/9
	3	0/9	—	0/9	**1/9**
VetMax™ ASFV Detection	2	**4/9**	—	—	**3/9**
	3	**1/9**	—	0/9	0/9
ASF System 1 (WOAH King et al. [15])	2	**2/9**	—	—	0/9
	3	0/9	—	0/9	0/9
RealPCR ASFV DNA	2	0/9	—	—	0/9
	3	0/9	—	**2/9**	0/9

Legend: — = true negative sample.

## Data Availability

The data are available on request from the corresponding author.

## References

[1] Andres G., Charro D., Matamoros T., Dillard R.S., Abrescia N.G.A. (2020). The cryo-EM structure of African swine fever virus unravels a unique architecture comprising two icosahedral protein capsids and two lipoprotein membranes. J. Biol. Chem..

[2] Alejo A., Matamoros T., Guerra M., Andres G. (2018). A Proteomic Atlas of the African Swine Fever Virus Particle. J. Virol..

[3] Penrith M.L., Vosloo W., Jori F., Bastos A.D. (2013). African swine fever virus eradication in Africa. Virus Res..

[4] Gallardo M.C., Reoyo A.T., Fernandez-Pinero J., Iglesias I., Munoz M.J., Arias M.L. (2015). African swine fever: A global view of the current challenge. Porc. Health Manag..

[5] Gallardo C., Nieto R., Soler A., Pelayo V., Fernandez-Pinero J., Markowska-Daniel I., Pridotkas G., Nurmoja I., Granta R., Simon A. (2015). Assessment of African Swine Fever Diagnostic Techniques as a Response to the Epidemic Outbreaks in Eastern European Union Countries: How To Improve Surveillance and Control Programs. J. Clin. Microbiol..

[6] Chenais E., Depner K., Guberti V., Dietze K., Viltrop A., Stahl K. (2019). Epidemiological considerations on African swine fever in Europe 2014-2018. Porc. Health Manag..

[7] Zhou X., Li N., Luo Y., Liu Y., Miao F., Chen T., Zhang S., Cao P., Li X., Tian K. (2018). Emergence of African Swine Fever in China, **2018**. Transbound Emerg. Dis..

[8] Gonzales W., Moreno C., Duran U., Henao N., Bencosme M., Lora P., Reyes R., Nunez R., De Gracia A., Perez A.M. (2021). African swine fever in the Dominican Republic. Transbound Emerg. Dis..

[9] Galindo I., Alonso C. (2017). African Swine Fever Virus: A Review. Viruses.

[10] Achenbach J.E., Gallardo C., Nieto-Pelegrin E., Rivera-Arroyo B., Degefa-Negi T., Arias M., Jenberie S., Mulisa D.D., Gizaw D., Gelaye E. (2017). Identification of a New Genotype of African Swine Fever Virus in Domestic Pigs from Ethiopia. Transbound Emerg. Dis..

[11] Bastos A.D., Penrith M.L., Cruciere C., Edrich J.L., Hutchings G., Roger F., Couacy-Hymann E., Thomson G.R. (2003). Genotyping field strains of African swine fever virus by partial p72 gene characterisation. Arch. Virol..

[12] Blome S., Franzke K., Beer M. (2020). African swine fever—A review of current knowledge. Virus Res..

[13] Broekhuijse M.L., Gaustad A.H., Bolarin Guillen A., Knol E.F. (2015). Efficient Boar Semen Production and Genetic Contribution: The Impact of Low-Dose Artificial Insemination on Fertility. Reprod. Domest. Anim..

[14] Tignon M., Gallardo C., Iscaro C., Hutet E., Van der Stede Y., Kolbasov D., De Mia G.M., Le Potier M.F., Bishop R.P., Arias M. (2011). Development and inter-laboratory validation study of an improved new real-time PCR assay with internal control for detection and laboratory diagnosis of African swine fever virus. J. Virol. Methods.

[15] King D.P., Reid S.M., Hutchings G.H., Grierson S.S., Wilkinson P.J., Dixon L.K., Bastos A.D., Drew T.W. (2003). Development of a TaqMan PCR assay with internal amplification control for the detection of African swine fever virus. J. Virol. Methods.

[16] Fernandez-Pinero J., Gallardo C., Elizalde M., Robles A., Gomez C., Bishop R., Heath L., Couacy-Hymann E., Fasina F.O., Pelayo V. (2013). Molecular diagnosis of African Swine Fever by a new real-time PCR using universal probe library. Transbound Emerg. Dis..

[17] Aguero M., Fernandez J., Romero L., Sanchez Mascaraque C., Arias M., Sanchez-Vizcaino J.M. (2003). Highly sensitive PCR assay for routine diagnosis of African swine fever virus in clinical samples. J. Clin. Microbiol..

[18] Schulz C., van der Poel W.H., Ponsart C., Cay A.B., Steinbach F., Zientara S., Beer M., Hoffmann B. (2015). European interlaboratory comparison of Schmallenberg virus (SBV) real-time RT-PCR detection in experimental and field samples: The method of extraction is critical for SBV RNA detection in semen. J. Vet. Diagn. Investig..

[19] Pikalo J., Carrau T., Deutschmann P., Fischer M., Schlottau K., Beer M., Blome S. (2022). Performance Characteristics of Real-Time PCRs for African Swine Fever Virus Genome Detection-Comparison of Twelve Kits to an OIE-Recommended Method. Viruses.

[20] Schoder M.E., Tignon M., Linden A., Vervaeke M., Cay A.B. (2020). Evaluation of seven commercial African swine fever virus detection kits and three Taq polymerases on 300 well-characterized field samples. J. Virol. Methods.

[21] WOAH Register of Diagnostic Kits Certified by the OIE as Validated as Fit for Purpose. https://www.woah.org/en/what-we-offer/veterinary-products/diagnostic-kits/the-register-of-diagnostic-kits/.

[22] Hoffmann B., Schulz C., Beer M. (2013). First detection of Schmallenberg virus RNA in bovine semen, Germany, 2012. Vet. Microbiol..

[23] Louwrier A., van der Valk A. (2001). Can sucrose affect polymerase chain reaction product formation?. Biotechnol. Lett..

[24] Zani L., Forth J.H., Forth L., Nurmoja I., Leidenberger S., Henke J., Carlson J., Breidenstein C., Viltrop A., Hoper D. (2018). Deletion at the 5′-end of Estonian ASFV strains associated with an attenuated phenotype. Sci. Rep..

[25] Friedrichs V., Reicks D., Hasenfuss T., Gerstenkorn E., Zimmerman J.J., Nelson E.A., Carrau T., Deutschmann P., Sehl-Ewert J., Roszyk H. (2022). Artificial Insemination as an Alternative Transmission Route for African Swine Fever Virus. Pathogens.

[26] Pikalo J., Deutschmann P., Fischer M., Roszyk H., Beer M., Blome S. (2021). African Swine Fever Laboratory Diagnosis-Lessons Learned from Recent Animal Trials. Pathogens.

[27] Mallik S. (2014). Identification Methods | Multilocus Enzyme Electrophoresis. Encyclopedia of Food Microbiology.

[28] Bland J.M., Altman D.G. (1986). Statistical methods for assessing agreement between two methods of clinical measurement. Lancet.

[29] Da Silva N., Zardoya R., Santurde G., Solana A., Castro J.M. (1995). Rapid and sensitive detection of the bovine viral diarrhea virus genome in semen. J. Virol. Methods.

[30] Reicks D.L., Muñoz-Zanzi C., Rossow K. (2006). Sampling of adult boars during early infection with porcine reproductive and respiratory syndrome virus for testing by polymerase chain reaction using a new blood collection technique (blood-swab method). JSHAP.

[31] Pepin B.J., Kittawornrat A., Liu F., Gauger P.C., Harmon K., Abate S., Main R., Garton C., Hargrove J., Rademacher C. (2015). Comparison of specimens for detection of porcine reproductive and respiratory syndrome virus infection in boar studs. Transbound Emerg. Dis..

[32] Nybo K. (2011). qPCR: Technical Replicate Variation. Biotechniques.

